# On-Chip Glucose Detection Based on Glucose Oxidase Immobilized on a Platinum-Modified, Gold Microband Electrode

**DOI:** 10.3390/bios11080249

**Published:** 2021-07-25

**Authors:** Julia Madden, Colm Barrett, Fathima R. Laffir, Michael Thompson, Paul Galvin, Alan O’ Riordan

**Affiliations:** 1 Tyndall National Institute, University College Cork, T12 R5CP Cork, Ireland; colm.barrett@tyndall.ie (C.B.); m.thompson@utoronto.ca (M.T.); paul.galvin@tyndall.ie (P.G.); 2 Bernal Institute, University of Limerick, V94 T9PX Limerick, Ireland; Fathima.Laffir@ul.ie; 3 Department of Chemistry, University of Toronto, Toronto, ON M5S 3H6, Canada

**Keywords:** microband, glucose detection, enzymatic, electrochemical, microfabrication, silicon

## Abstract

We report the microfabrication and characterization of gold microband electrodes on silicon using standard microfabrication methods, i.e., lithography and etching techniques. A two-step electrodeposition process was carried out using the on-chip platinum reference and gold counter electrodes, thus incorporating glucose oxidase onto a platinum-modified, gold microband electrode with an o-phenylenediamine and ß-cyclodextrin mixture. The as-fabricated electrodes were studied using optical microscopy, scanning electron microscopy, and atomic force microscopy. The two-step electrodeposition process was conducted in low sample volumes (50 µL) of both solutions required for biosensor construction. Cyclic voltammetry and electrochemical impedance spectroscopy were utilised for electrochemical characterization at each stage of the deposition process. The enzymatic-based microband biosensor demonstrated a linear response to glucose from 2.5–15 mM, using both linear sweep voltammetry and chronoamperometric measurements in buffer-based solutions. The biosensor performance was examined in 30 µL volumes of fetal bovine serum. Whilst a reduction in the sensor sensitivity was evident within 100% serum samples (compared to buffer media), the sensor demonstrated linear glucose detection with increasing glucose concentrations (5–17 mM).

## 1. Introduction

The fabrication of silicon-based electrodes is valuable for the development of miniaturized electrochemical biosensors, as various electrode designs, dimensions, and configurations can be fabricated on a compact electroanalytical device [1,2]. Micro-and nanoscale electrodes have become increasingly popular for the advancement of electrochemical-based biosensors because of their lower detection limits, higher signal-to-noise ratio, and ability to achieve steady-state currents [3,4,5]. Electrochemical glucose sensors continue to be of significant interest due to their applications in a variety of fields, including human health [6], animal health [7], bioprocess monitoring [8], and monitoring in the food and beverage industries [9,10]. In the present work, we describe a single gold band electrode that is 45 µm in length, 1 µm in width, and has a height of ~80–90 nm. This specific type of electrode is commonly referred to as an ultramicroelectrode, as it has at least one dimension that is less than a number of micrometers [11,12]. Ultramicroelectrodes (UME’s) have been developed for several applications, including heavy metal detection [13], single-cell electrochemical measurements [14], glucose [15], DNA [16], bacteria [17], and virus detection [18]. Previous work carried out on single gold nanowires, fabricated in a similar manner to those described within this article [19], demonstrated glucose detection using an external redox reagent (ferrocene monocarboxylic acid) and glucose oxidase free in solution. The removal of redox mediators can simplify device fabrication, reduce costs, and eliminate the potential toxicity problems associated with next-generation electrochemical bio-sensors [20]. In the case of glucose detection, one potential advantage of using ultra-microelectrodes is that they benefit from radial mass transport, and the sensors developed herein are not limited by local oxygen diffusion at the electrode surface, unlike their larger macro-electrode analogues [21]. Pt-Black (Pt-B) was electrodeposited on to the band electrode, as Pt-B nanostructured layers are well known to offer a catalytic surface for the oxidation of hydrogen peroxide [22], in addition to increasing the active surface area for the electro-polymerized mixture containing GOx. The use of o-PD has been widely investigated for both the immobilization of glucose oxidase on to electrodes [23,24,25] and its ability to prevent interfering species, such as ascorbic acid [26] and uric acid [27], from reaching the electrode surface. This permselective layer has previously been used to immobilize GOx on to electrodes with dimensions in the nano to micrometer scale [28] and more recently has been applied for diagnostic [29], wearable [30], and in vivo-based biosensors [31]. Whilst studies have explored both mediator-free glucose detection and the use of o-PD modified microelectrodes towards glucose sensing, here, we demonstrate the amperometric deposition of a mixture of ß-cyclodextrin, o-phenylenediamine (o-PD), and glucose oxidase on to an ultramicroband electrode using the on-chip Pt reference (RE) and Au counter (CE) electrodes. This modification process allowed for the use of lower sample volumes (hence, the number of units of enzymes) during the modification stages. Sulphonated β-cyclodextrin was incorporated into the mixture, as it has been previously shown to further mitigate against interference effects in the presence of an electropolymer coating. This is a result of the 7–11–SO_3_^−^ charges present on sulphonated β-cyclodextrin, which repel ascorbate anions [32,33]. Linear sweep voltammetry (LSV) and chronoamperometry techniques were used to demonstrate the on-chip glucose detection, using the on-device Pt RE and Au CE electrodes. The chip developed herein demonstrated the analysis in relatively low sample volumes of 30–50 µL (applicable to the well size in the chip holder). The sensor demonstrated detection in phosphate-based buffer solutions (2.5 mM–15 mM), as well as the measurement of glucose in the presence of potential interfering species. Sensor performance was finally examined for the potential real applications in fetal bovine serum spiked with known concentrations of glucose, within a linear range appropriate to physiological glucose concentrations (~4–7 mM) [34].

## 2. Materials and Methods

### 2.1. Materials

Ferrocenemonocarboxylic acid (FcCOOH), phosphate buffered saline (PBS), o-phenylenediamine (o-PD), β-cyclodextrin, sodium sulphate, sodium phosphate monobasic, sodium phosphate dibasic, hexachloroplatanic acid, lead acetate trihydrate, hydrogen peroxide (H_2_O_2_) 30 wt.%, D-(+)-glucose, D-(−)-fructose, D-(+)-mannose, sulphuric acid, L-ascorbic acid, salicylic acid, and uric acid were purchased from Sigma Aldrich Ltd. Glucose oxidase (GOx, E.C. 1.1.3.4) from Aspergillus niger (≤42.7 U/mg) was purchased from Sekisui Diagnostics Ltd., Maidstone, UK. All reagents were prepared in 18.2 MΩ deionized water (ELGAPurelab, High Wycombe, UK). Fetal bovine serum was purchased from Thermofisher Scientific, Waltham, Massachusetts, United States.

### 2.2. Apparatus and Instrumentation

An Autolab potentiostat PGSTAT302N electrochemical workstation (Metrohm Ltd., Utrecht, The Netherlands) was used to conduct electrochemical analysis. Unless otherwise stated, all electrochemical characterisations and modifications were carried out using the on-chip CE and RE. A chip holder was machined using a Bridgeport Hardinge VMC 480 CNC machine to connect the chip to the potentiostat (see Supplementary Materials Figure S1A). The silicon chip was fabricated to contain six individually addressable, single microband working electrodes (WE’s); a gold macroband electrode was used as a CE, and a platinum macroband electrode was employed as a pseudo-RE (see Supplementary Materials Figure S1B,C). Microscopy images were acquired using an Olympus camera and epifluorescence microscope (Model BX51 TRF SN 5M18343). Scanning electron microscopy (SEM) images were taken with a Zeiss Supra 40 SEM. Atomic Force Microscopy (AFM) was carried out with a Veeco Dimension 3100 atomic force microscope in tapping mode using commercial Olympus probes (Oxford Instruments Asylum Research, High Wycombe, UK). An area of 5 µm × 5 µm was investigated at a scan rate of 0.7 Hz. Background plane subtraction was applied to the images. X-ray Photoelectron Spectroscopy (XPS) was carried out at the University of Limerick using a Kratos AXIS ultra-spectrometer equipped with a mono Al Kα X-ray gun. Survey and high-resolution scans were recorded with a 1 eV and 0.05 eV resolution, respectively. The Freestyle Optimum Neo One glucometer and test strips (Abbott) were used to confirm the glucose concentrations in un-spiked fetal bovine serum. All recorded measurements were imported into OriginPro 2016 for data analysis.

### 2.3. Sensor Preparation

**Scheme 1:**
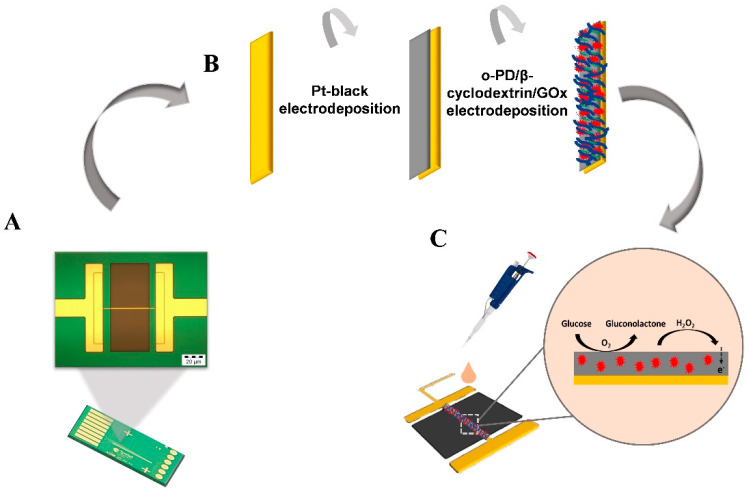
(**A**) The chip platform with a magnified image of the single microband electrode (50× microscopy image), (**B**) the modification process for enzyme immobilization on to the single microband electrode, and (**C**) schematic of the glucose detection mechanism at the electrode solution interface.

Single microband electrodes were fabricated using a standard silicon microfabrication approach (see Supplementary Materials Figure S2), including lithography, deposition, and etching on silicon wafers, as described previously [35,36,37]. Prior to use, the chip (see Scheme 1A) was cleaned by ultrasonication in isopropanol, acetone, and deionized water for 5 min each. The modification process (see Scheme 1B) was similar to that described by Barrett et al., 2019 [33]. A platinum-black layer was deposited via amperometry from a solution containing 15 mM hexachloroplatinic acid and 2.1 mM lead acetate, at −0.75 V against the on-chip platinum RE for 10 s. The o-PD/β-cyclodextrin/GOx mixture was then electrodeposited at a potential of 0.35 V versus the on-chip Pt RE for 1200 s. Both electrochemical chronoamperograms can be viewed in the Supplementary Materials (Figure S3A,B). Electro-polymerization was carried out from a solution consisting of a mixture of 5 mM o-PD, 2.5 mM sulphonated-β-cyclodextrin, 5 mM sodium sulphate, and 10 mg/mL of glucose oxidase in a 50 mM phosphate buffer (PB) (sodium phosphate monobasic and sodium phosphate dibasic) solution at pH 7.4. Following electrode modification, WE’s were rinsed with 50 mM PB to remove any unbound enzymes from the microband. The chips were then stored in 50 mM PB (pH 7.4) overnight at 4 °C prior to measurements to allow the biosensor to stabilize.

### 2.4. Sensor Characterisation

Cyclic Voltammetry (CV) in 0.1 M H_2_SO_4_ was carried out on the bare gold electrode to assess for gold oxide formation and removal. CV and electrochemical impedance spectroscopy (EIS) were carried out following each surface modification in 1 mM FcCOOH prepared in 10 mM PBS. The charge transfer process, occurring at the electrode-solution interface, was measured using EIS following each electrodeposition. EIS was carried out at a frequency range of 100 mHz to 100 kHz and a potential of 200 mV. o-PD concentration (mM) was examined against a 0.5 mM glucose addition at the electrode surface (see Supplementary Materials Figure S4). SEM images were obtained to visualize the changes in the surface morphology between the bare Au, Pt-B, and o-PD/β-cyclodextrin/GOx microband surfaces. Finally, XPS measurements were conducted on electrode modifications on a macro-Au band electrode (90 µm × 7 mm), as the dimensions of the microband electrodes were unsuitable to the resolution required for XPS analysis (see Supplementary Materials Figure S5). These modifications could not be conducted with an on-chip RE and CE, thus, were conducted with a Pt wire (counter) and an Ag/AgCl reference. In this instance, the potential of both the depositions described in Section 2.3 were adjusted by +0.15 V, owing to the potential shift occurring when using the Ag/AgCl external RE in place of the Pt-pseudo-RE.

### 2.5. Electrochemical Measurements

In this study, glucose detection is quantified from enzymatically produced hydrogen peroxide (Scheme 1C). For this reason, the o-PD/β-cyclodextrin surface modification was tested in various concentrations of hydrogen peroxide to ensure that H_2_O_2_ detection was possible at the modified UME, in the absence of the GOx enzyme. LSV and chronoamperometric techniques were utilised to measure the response of glucose additions at the o-PD/β-cyclodextrin/GOx electrode surface in 50 mM PB (pH 7.4) against the on-chip RE and CE. Chronoamperometry was used to assess sensor performance in the presence of common interfering species. Preliminary short-term operational stability was examined by completing a calibration on the same sensor, on two consecutive days, after preparing the biosensor. Short-term storage tests were carried out by measuring a 2.5 mM glucose concentration after 4 days of storage at 4 °C, for comparison with a separate chip response to a 2.5 mM glucose concentration measured after 1 day of storage. Lastly, single-sample measurements were conducted with 100% fetal bovine serum, spiked with known glucose concentrations, to assess the on-chip sensor response in a bio-fluid-based environment. Additionally, 30 µL aliquots were added to the well, and chronoamperometry was applied for 70 s at 0.25 V. The mean current density, over a sampling time of 40 to 70 s, was plotted against the unknown glucose concentration (U), as well as U + 2mM, 4 mM, 6 mM, 8 mM, 10 mM, and 12 mM glucose additions. The standard addition technique was used to approximate the concentration of the unknown sample. A background subtraction signal could not be obtained for the calibration, as FBS with zero glucose concentration was unavailable. The FBS was spiked with low volumes of glucose, prepared in 50 mM PB, to maintain the media close to 100% of the original matrix. Finally, the un-spiked FBS was tested with a glucometer to obtain the unknown glucose concentration.

## 3. Results and Discussion

### 3.1. Characterisation

**Figure 1:**
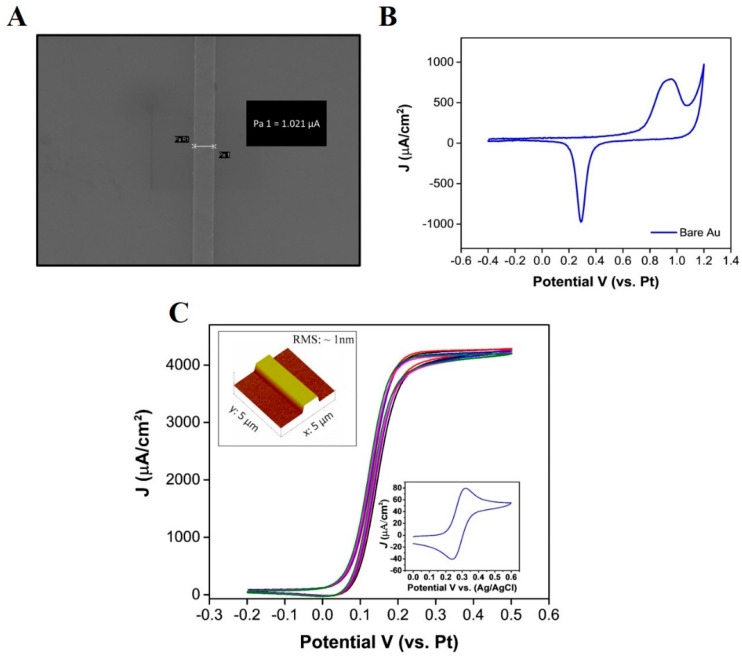
(**A**) SEM image showing a width measurement of 1.021 µm, (**B**) cyclic voltammogram obtained in 0.1 M H_2_SO_4_ at a scan rate of 50 mVs^−1^, and (**C**) cyclic voltammogram obtained in 1 mM FcCOOH for *n* = 5 electrodes on one chip (RSD < 0.1%), (inset graph shows a cyclic voltammogram obtained at a macro-Au band electrode), inset figure depicts a 3D atomic force microscopy image of a 5 µm × 5 µm area at a scan rate of 0.7 Hz (Background plane subtraction was applied to the image).

Figure 1A displays a SEM image of the band electrode, with a line measurement of ~1.021 µm across the width of the band. A CV in sulfuric acid was used to confirm the presence of a gold layer by showing the characteristic formation of surface oxides (anodic sweep) and their removal (cathodic sweep) [38]. Figure 1B shows a typical voltammogram, obtained at the bare Au electrode; an oxidation peak can be seen at 0.9 V versus the on-chip platinum RE electrode. This peak represents the formation of the gold oxide (Au_2_O_3_) layers. During the reverse sweep, a peak can be seen at 0.29 V, resulting from the removal of the gold oxide layers. An AFM characterization was also undertaken, and a typical micrograph (5-micron size) was obtained at the bare gold band electrode (see inset of Figure 1C). This data confirmed the electrode width to be ~1 µm and the height measured was ~90 nm. A root-mean-square (RMS) roughness value of less than 1 nm was measured for the e-beam evaporated gold electrodes. CV was also used to examine microelectrode behaviour in the presence of the electroactive probe (1mM FcCOOH). The resultant CVs exhibit a quasi steady-state behaviour (Figure 1C), arising from radial diffusion mass transport to the electrode, indicative of ultramicroband electrodes. The single band sensors demonstrated excellent intra-chip reproducibility, as a RSD of less than 0.1% was determined between both the steady state oxidation and reduction currents for *n* = 5 WE’s in 1 mM FcCOOH, at a scan rate of 50 mVs^−1^. This also indicated that the on-chip RE and CE were effective for electrochemical measurements. The current generated (2 nA) confirms that electron transfer is taking place at the ultra-microband electrode. Furthermore, it is clear that the passivation is functional, as it inhibits an electrochemical response at other metallised regions of the chip. For comparison purposes, a CV was also carried out on a macro-Au band electrode (dimensions 90 µm × 7 mm) under the same conditions (50 mVs^−1^ scan rate) versus Ag/AgCl; a peak shaped CV was produced, depicting planar diffusion to the larger band electrode (see inset of Figure 1C). The data were normalised to current density using the approximate geometric area (macro-electrode: $6.3\times 10^{-3}\;{\mathrm{cm}}^{2}$, microelectrode: $5\times 10^{-7}\;{\mathrm{cm}}^{2}$) of both electrodes, which were determined from the dimensional information (macro-electrode: 90 µm×7 mm, microband electrode: 90 nm×1 µm×45 µm) obtained with the SEM, AFM, and fabrication designs to allow direct comparison. As expected, higher current densities were observed at the microband electrode, due to the radial diffusion regime, in comparison to the macroelectrode, demonstrating the higher sensitivity associated with microelectrodes.

**Figure 2:**
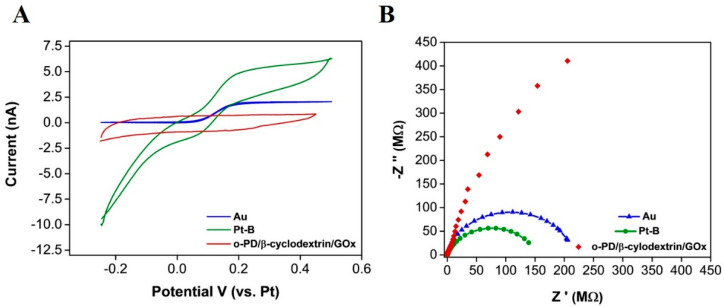
(**A**) CV in 1 mM FcCOOH at 50 mVs^−1^, at the bare gold (blue), Pt-B modified (green) and o-PD/β-cyclodextrin/GOx modified (red) microband electrode and (**B**) Nyquist plots of EIS measurements at a bare gold (blue) [Rct 217 MΩ], Pt-B modified (green) [Rct 151 MΩ] and o-PD/β-cyclodextrin/GOx modified [unfitted data] (red).

Following each step of the fabrication process, a number of techniques were employed to understand the changes to the surface properties. CV was used to obtain voltammograms in the 1 mM FcCOOH redox reagent after each modification (Figure 2A). An increase in the oxidation current of around 3 nA was seen between the Au (Blue) and Pt –modified (green) surface (at 0.2 V), resulting from the enhanced electroactive area of the Pt-B modification. A clear decrease in both oxidation and reduction currents is visible after the o-PD/β-cyclodextrin/GOx deposition, which can be attributed to the formation of the o-PD layer [39]. EIS was used to assess the change in charge transfer after each electrodeposition (Figure 2B). Nyquist plots can include a semi-circular region and a linear part. At higher frequencies, the semicircular region relates to the electron transfer limited process. The diameter of the semi-circle corresponds to the electron transfer resistance (R_ct_). The R_ct_ decreased from 217 MΩ at the bare Au surface to 151 MΩ, following the introduction of an enhanced surface area Pt-B electrodeposition. Following o-PD/β-cyclodextrin/GOx deposition, under the same parameters, a full semi-circle was not obtained for an electrochemical circle fit. However, there is a clear change in impendence behaviour; this final layer is preventing the electron transfer of the redox probe. A diffusion-controlled effect was not visible, as the electrodes were diffusionally independent.

**Figure 3:**
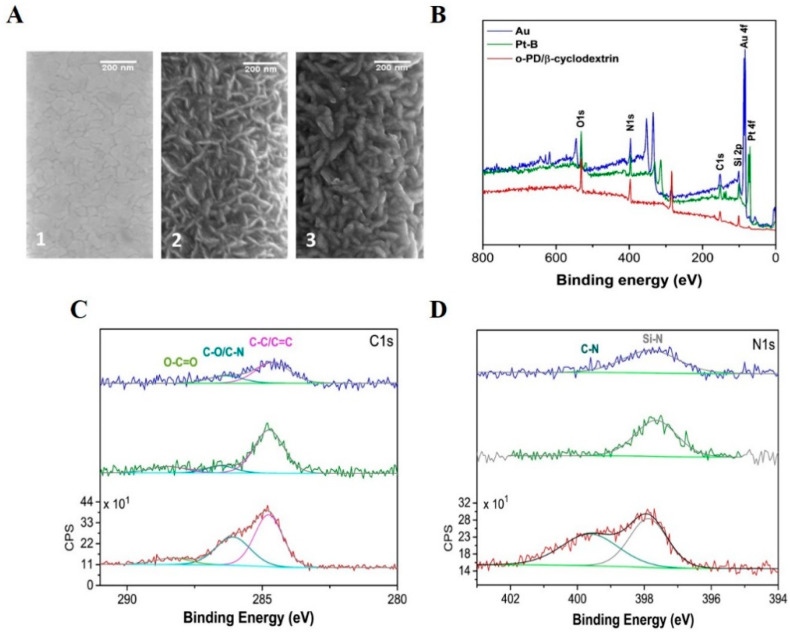
(**A**) Scanning electron microscopy images taken at (1) bare Au electrode surface, (2) Pt-B modified electrode surface, and (3) Pt-B/o-PD/ β-cyclodextrin/GOx modified electrode surface; (**B**) XPS survey scan of the bare Au (blue), as modified Pt-B (green), and as modified Au/Pt-B/o-PD/β-cyclodextrin (red); (**C**) high-resolution scans of the C1s peaks, and (**D**) high-resolution scans of the N1s peaks.

The SEM images display the presence of a nanodentritic Pt-B coating on to the 1 µm band electrode, with a clear change in the surface morphology (see Figure 3A 1,A 2). Following the o-PD/β-cyclodextrin/GOx deposition, subtle changes to dendrite thickness were observed; however, this was not quantifiable (see Figure 3A 3). In order to demonstrate the effectiveness of the electrodepositions as a modification method, a larger macroband electrode was modified amperometrically, with both Pt-B and o-PD/β-cyclodextrin. The XPS surveys obtained at each electrode modification demonstrate a change in the surface composition. Firstly, Figure 3B depicts the introduction of a platinum peak Pt 4f post Pt-B deposition (green layer). The atomic percentage of Pt decreased to 0.2%, following the electrodeposition of the o-PD/β-cyclodextrin layer. XPS probes the upper 10 nm of the surface; the decrease in Pt signal intensity occurs relative to the introduction of the C-N signal from o-phenylenediamine at 286.1 eV in the C1s high-resolution spectra and at 399.6 eV in the N1s high-resolution spectra (Figure 3C,D), indicating that the o-PD layer has successfully electrodeposited [33,40]. The XPS measurements confirmed the presence of both Pt-B and o-PD on a larger gold band electrode (fabricated on the silicon substrate). The quantification of each electrode modification is tabulated in Table S1 of the Supplementary Materials.

### 3.2. Electrochemical Measurements

**Figure 4:**
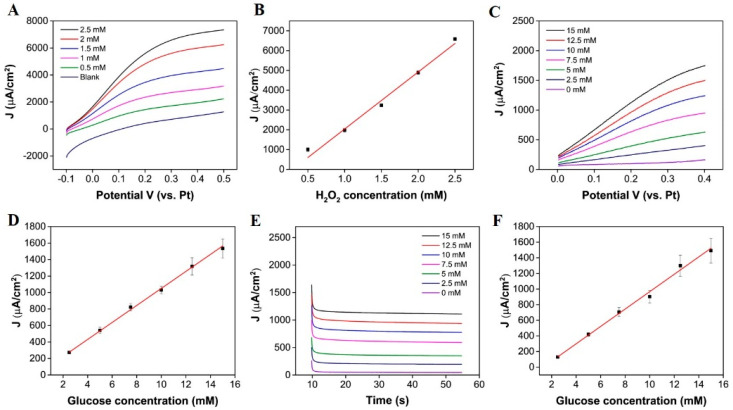
(**A**) LSV obtained at a scan rate of 50 mVs^−1^ in increasing concentrations of H_2_O_2_ at the Au/Pt-B/o-PD/β-cyclodextrin surface, (**B**) corresponding calibration obtained from background subtracted data at 0.25 V, (**C**) LSV obtained at a scan rate of 50 mVs^−1^ from 0 to 0.4 V in increasing glucose concentrations in 50 mM PB solution (pH 7.4), (**D**) corresponding calibration obtained from background subtracted data at 0.25 V, (**E**) i-t curves for the chronoamperometric response of the biosensor to increasing glucose concentrations in 50 mM PB (pH 7.4), and (**F**) the resulting calibration curve.

As previously mentioned, in this work, glucose is measured from enzymatically produced hydrogen peroxide; whilst it was expected that H_2_O_2_ can permeate o-PD modification layers [41], we confirmed this with LSV in various concentrations of H_2_O_2_. Figure 4A displays LSV measurements, obtained in increasing concentrations of H_2_O_2_ at the Au/Pt-B/o-PD/β-cyclodextrin surface. An increasing faradaic current was observed with increasing H_2_O_2_ concentrations. The current was converted to current density using an approximate geometric area of the microband after sensor preparation ($1.35\times 10^{-6}{\;\mathrm{cm}}^{2})$, which was determined using dimensional information (3 µm×45 µm); (the width was approximated using AFM see Supplementary Materials Figure S6). These results show that H_2_O_2_ can penetrate through the modification layer to reach the underlying platinum-modified surface and undergo electrochemical oxidation. A plot of peak current density versus H_2_O_2_ concentration is presented in Figure 4B (the error bars represent the standard error between the mean peak current densities measured for *n* = 3 chips). The plot exhibits good linearity (coefficient of deviation (COD) R^2^ = 0.993), indicating that the sensor response to hydrogen peroxide is linear within this concentration range. The slope of the line was used to obtain a sensitivity value of 2882 µA mM^−1^ cm^−2^. The theoretical LOD was determined to be 17 µM, using the blank method, as described in [42]. Both LSV and chronoamperometry were employed for on-chip glucose measurements in six pre-determined aliquots of glucose (2.5 mM to 15 mM), made up in 50 mM PB (pH 7.4). Figure 4C displays the LSV measurements obtained in increasing concentrations of glucose; an increase in current density is seen with increasing glucose concentrations (2.5 mM to 15 mM (COD R^2^ = 0.998), see Figure 4D (the error bars represent the standard error between the mean current density of *n* = 3 chips). Using LSV, the sensitivity determined was 103.11 µA mM^−1^ cm^−2^, and the theoretical LOD was determined to be 1.42 mM. Figure 4E displays the chronoamperometric response of the microband at 0.25 V for 45 s measurements in the same aliquots of glucose. The biosensor reached a steady-state response within 20 s of applying a potential. A plot of current density versus glucose concentration is presented in Figure 4F (the error bars represent the standard error between the mean current density for *n* = 3 chips and the mean current density of each sensor was determined from 30 data points, obtained after a 20s sampling time), where a linear increase in current density is seen with increasing glucose concentrations (COD R^2^ = 0.997). The sensitivity was determined to be 111.21 µA mM^−1^ cm^−2^ for the chronoamperometric method with a theoretical LOD of 0.79 mM.

### 3.3. Selectivity, Short-Term Stability and Serum Analysis

**Figure 5:**
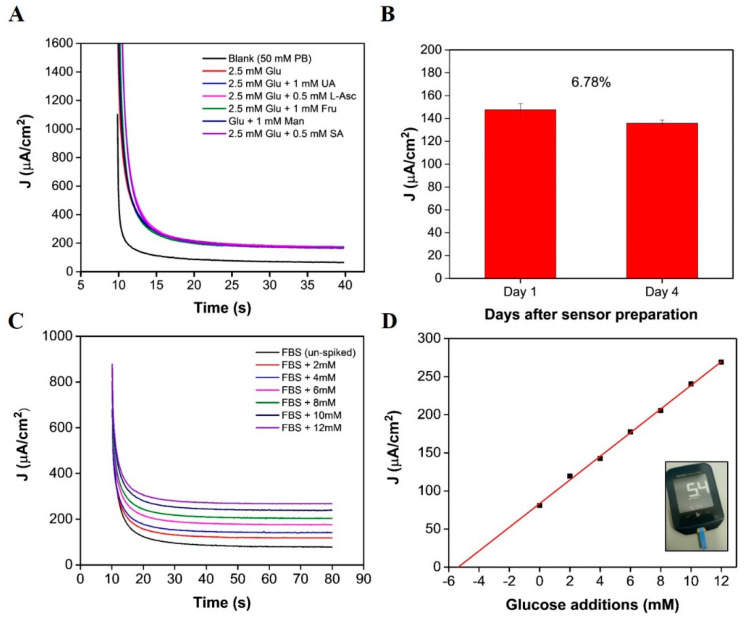
(**A**) Chronoamperometric response of the on-chip biosensor to a blank solution (50 mM PB, pH 7.4), to 2.5 mM glucose and to separate aliquots of 2.5 mM glucose + other potential interfering species (L-ascorbic acid (L-A) fructose (Fru), mannose (Man), uric acid (UA) and salicylic acid (SA); (**B**) bar chart depicting the 2.5 mM concentration measured after 1 day of storage and a 2.5 mM concentration measured 4 days after storage (error bars represent std. dev between 50 data points obtained from 20–40 s current sampling time of measurement); (**C**) chronoamperometric response of the sensor in 100% fetal bovine serum spiked with known concentrations of glucose; (**D**) corresponding standard addition for subfigure (**C**) (inset image shows 1 of 10 measurements obtained in FBS).

Chronoamperometry was applied for both interference and real sample analysis. Figure 5A displays the i-t curves obtained for measurements in a 2.5 mM glucose concentration, in addition to a 2.5 mM glucose concentration containing 0.5 mM L-ascorbic acid, 1 mM fructose, 1 mM maltose, 1mM uric acid, or 0.5 mM salicylic acid. Current densities obtained at a sampling time of 20 to 40 s were background subtracted and plotted as a bar chart (see Supplementary Materials Figure S7A). The error bars represent the standard deviation between 50 data points, obtained between the 20 to 40 s sampling time. The interference effect was also calculated using the following equation: $\left[\frac{I_{glucose}}{I_{interference}}\right]*100$ and response changes are tabulated in the Supplementary Materials (see Table S2). Preliminary short-term stability assessments demonstrate that the sensor could be re-used (see supplementary Figure S7B); a calibration was obtained 1 day after sensor preparation and again 2 days after sensor preparation. Excellent sensor recovery values were determined for three concentrations: 103.4%, 102.0%, and 98.07%, and satisfactory values were determined for the remaining three concentrations: 88.7%, 87.2%, and 87%. Biosensor performance, after 4 days of storage, was assessed in a 2.5 mM glucose concentration. The sensor demonstrated a sensor recovery value of 93.22% (see Figure 5B), indicating that the enzyme had remained stable over a short time period. For both sets of preliminary stability data, the biosensor demonstrated good short-term stability; however, it must be considered that this is not representative of a comprehensive stability study. Further investigation is necessary to understand the sensor behaviour over time and to determine the long-term storage capabilities [43,44]. In this study, as a potential proof of concept, the ultra-microelectrode response was measured in fetal bovine serum to evaluate the sensor’s performance in an un-diluted biological serum. The chronoamperometric response in a solution of FBS was measured for 70 s at 0.25 V. The serum aliquots (of equal volume) were subsequently spiked with low volume additions from a standard of known concentration. The resulting i-t curves show an increase in signal response for increasing glucose concentrations (Figure 5C). Using linear regression, the x-intercept was determined to be −5.49, indicating a glucose concentration of 5.49 mM (Figure 5D) [45]. A commercial glucometer (Abbott Freestyle Optimum Neo) confirmed a concentration of 5.07 ± 0.38 mM for *n* = 10 samples. The increase in concentration, determined using the standard addition approach, could be a result of the background signal of the FBS. Measurements conducted in fetal bovine serum showed a clear decrease in signal response (see Figure 5C,D) in comparison to the calibration obtained in buffer-based solution (see Figure 4E,F). Similar sensitivity changes, owing to a change of matrix, have been reported by Bollella et al., whereby, a microneedle sensor sensitivity determined in buffer was 1473 μA cm^−2^ mM^−1^ and analysis in human serum revealed a sensitivity of 180 ±9 μA cm^−2^ mM^−1^ [46]. The composition of fetal bovine serum is more complex than that of buffer media, as proteins, growth factors, hormones, and electrolytes are present. We suggest that the reduction in the sensors sensitivity could be a result of the adsorption or precipitation of these agents to the ultramicroelectrode and/or the on-chip RE and CE electrodes. Despite the decrease in sensor sensitivity, a linear response was observed for increasing glucose concentrations (see Figure 5D) up to 17 mM, taking into account the original glucose concentration of FBS.

Table 1 presents a summary of the enzymatic-based glucose biosensors, also constructed on miniaturized electrodes, for comparison with the microband presented here. This work offers an ultramicroband electrode capable of measuring glucose concentrations on-chip, in comparison to many devices incorporating the conventional electrochemical set up with an external RE and CE. The operating potential is low, owing to the use of the pseudo-reference electrode. The linear detection range demonstrates the potential for sensing in bio-fluids such as blood, interstitial fluid, and saliva [47]. The high-sensitivity values determined for both the LSV and chronoamperometric techniques are comparable, or higher, than microelectrodes reported with a similar WE area.

## 4. Conclusions and Future Perspectives

Understanding the electrochemical behaviour of miniaturized electrodes on devices is becoming increasingly important as advances continue towards integrating microelectrodes on wearable, surgical, and other medical devices. In summary, we have demonstrated a simple two-step electrochemical modification to a single gold microband electrode using the on-chip Pt RE and Au CE. SEM confirmed a change in the surface morphology following each electrodeposition step. The electrochemical characteristics of the microbands were analysed in 1 mM FcCOOH, with EIS data demonstrating a clear change in the R_ct_ following each electrode modification. The XPS measurements were obtained on a macro-Au band following both the Pt electrodeposition and the o-PD/β-cyclodextrin electrodeposition, as the microband dimensions were not suitable for the XPS spot size requirements. The electro-polymerization of the o-PD/β-cyclodextrin/GOx layer was successful in immobilizing the enzyme on the surface of the microelectrode, resulting in glucose detection in buffer-based solutions. Both the chronoamperometry and LSV methods employed for glucose detection at the UME demonstrated linear detection for six predetermined aliquots of glucose (2.5 mM to 15 mM). Chronoamperometric measurements carried out in 100% fetal bovine serum showed a reduction in signal response in comparison to measurements carried out in phosphate-based buffers. Even so, the UME demonstrated a linear response to increasing glucose additions, from approximately 5 mM to 17 mM, showing their potential for analysis in more complex media environments. Whilst the linear dynamic range does not fulfil the entire range required for blood glucose measurements, there is clear potential for other bio-fluids, diluted media, and other target analytes. Future work on this platform should focus on the optimization of electrode materials within target media, in addition to testing electrodes over sustained periods to understand the long-term stability and monitoring capabilities of these miniaturized electrodes.

## Figures and Tables

**Table 1:** A comparison of the on-chip ultramicroelectrode glucose sensor, presented here with other reagent-free miniaturized enzymatic glucose biosensors, constructed towards various biomedical sensing applications.

Electrode Material *	Linear Range	Operating Potential	Enzyme Immobilization	Sensitivity	Real Sample	Reference
CFM/Pt-GOx(0.5%)-GA	0–4 mM	0.7 V vs. Ag/AgCl (external RE)	Covalent	0.697 nA mM^−1^ cm^−2^	Brain slices	[48]
CF-PEDOT-GOx	0.5–15 mM	−0.65 V vs. Ag/AgCl (external RE)	Entrapment	8.5 μA mM^−1^ cm^−2^	-	[49]
Pt/PEDGE/BSA-GOx	0–5 mM	0.85 V vs. Ag/AgCl (external RE)	Covalent	20.72 pA mM^−1^	Intracellular	[50]
Pt-B electrode	0.2–43.5 mM	0.7 V vs. Ag/AgCl (on-device)	Free in solution	-	Serum samples	[51]
Pt/PAA/CS/AuNPs/GOx	0.017–0.81 mM	−0.4 V to 0.4 V to vs. Pt (on-device)	Layer-by-layer assembly	61.4 μA mM^−1^ cm^−2^	Saliva	[52]
Silver/Nafion/GA-GOx	0–2 mM	0.05 V vs. Ag/AgCl (external electrode)	Covalent	1.73 μA mM^−1^	-	[53]
Pt/GA-BSA-GOx/PU/Nafion	0–11 mM	0.5 V vs. IrOx (on-device)	Covalent	1.51 nA mM^−1^	Intradermal	[54]
GOx-β-cyclodextrin-o-PD/Pt-B/Au	2.5–15 mM	0.25 V vs. Pt (on-device)	Entrapment	111.21 µA mM^−1^ cm^−2^ (Buffer), 15 µA mM^−1^ cm^−2^ (Serum)	Fetal bovine serum	This work

* Abbreviations: CFM: carbon fibre microelectrode, Pt: platinum, GOx: glucose oxidase, GA: glutaraldehyde, CF: carbon fibre, PEDOT: poly (3, 4-ethylenedioxythiophene), PEDGE: poly (ethylene glycol) diglycidyl ether, BSA: bovine serum albumin, PAA: poly (allylamine), CS: chitosan, AuNPs: gold nanoparticles, and PU: polyurethane.

## Data Availability

Data is contained within the article.

## Supplementary Materials

The following are available online at https://www.mdpi.com/article/10.3390/bios11080249/s1, Figure S1 (A) Custom made chip holder, (B) image of chip showing the on-chip counter (Au) and pseudo-reference (Pt) electrodes, the interconnection tracks and (C) the single microband electrodes. Figure S2 Schematic of the sensor fabrication process. Figure S3. (A) Typical amperometric deposition of Pt-B at −0.75V versus the on-chip platinum reference electrode for 10 seconds with a quiet time of 2 seconds (inset shows microscopy image post electrodeposition) (B) Typical amperometric deposition of 5 mM o-phenylenediamine with 2.5 mM β-cyclodextrin and 10 mg/ml of Glucose Oxidase for 20 min. Figure S4. Current response for a 0.5 mM glucose concentration at the Au/Pt-B/o-PD/β-cyclodextrin surface where the o-PD concentration was varied (1.25 mM, 2.5 mM and 5 mM) whilst maintaining GOx at 10 mg/ml and the β-cyclodextrin at 2.5 mM in 50 mM PB (pH 7.4). Figure S5. Minimum spot size for XPS analysis on a planar macroscopic band electrode. Table S1. Quantification from XPS survey spectra at each modification. Figure S6. AFM section analysis of the Pt-B/o-PD/ β-cyclodextrin/GOx modified electrode surface. Table S2. Table depicting change in current in the presence of interfering species. Figure S7. (A) Bar chart depicting the background subtracted currents of a 2.5 mM glucose solution and that of a 2.5 mM glucose solution with other potential interfering species (B) A calibration obtained at the same sensor at Day 1 and again at Day 2 after sensor preparation.

## Rights Retention Statement

This publication has emanated from research supported, in part, by a grant from Science Foundation Ireland (under Grant numbers 12/RC/2289 and 12/RC/2289-P2) and, in part, from a research grant from Science Foundation Ireland and the Department of Agriculture, Food and Marine, on behalf of the Government of Ireland (under the Grant 16/RC/3835 (VistaMilk)). For the purpose of Open Access, the author has applied a CC BY public copyright license to any Author Accepted Manuscript version arising from this submission.
