# Supplementary material for: On-Chip Glucose Detection Based on Glucose Oxidase Immobilized on a Platinum-Modified, Gold Microband Electrode
Source: Biosensors (Basel). 2021 Jul 25;11(8):249. doi: 10.3390/bios11080249 (PMC8392376; doi:10.3390/bios11080249)
Supplement: Supplementary file 1 [file biosensors-11-00249-s001.zip › biosensors-1233080-supplementary.pdf]

Supplementary Materials for

On-Chip Glucose Detection Based on Glucose Oxidase Immobilized on a Platinum-Modified, Gold Microband Electrode

Julia Madden [1,*], Colm Barrett [1], Fathima Laffir [2] Michael Thompson [1, 3], Paul Galvin [1] and Alan O' Riordan [1,*]

[1] Tyndall National Institute, University College Cork, Cork, T12R5CP, Ireland; colm.barrett@tyndall.ie (C.B.); m.thompson@utoronto.ca (M.T.); paul.galvin@tyndall.ie (P.G.)
[2] Bernal Institute, University of Limerick, Limerick, V94 T9PX, Ireland
[3] Department of Chemistry, University of Toronto, Toronto, ON M5S 3H6, Canada
* Correspondence: julia.madden@tyndall.ie (J.M.); alan.oriordan@tyndall.ie (A.O.R.)

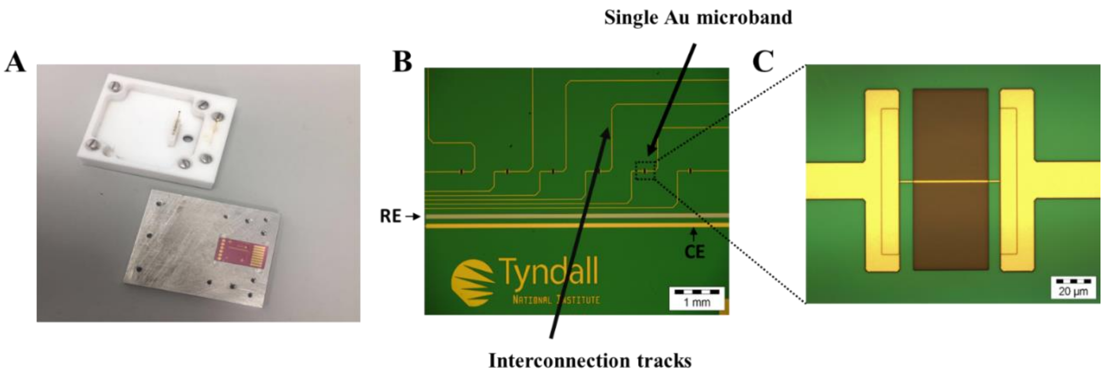

Figure S1. A) Custom made chip holder, B) image of chip showing the on-chip counter (Au) and pseudo-reference (Pt) electrodes, the interconnection tracks and C) the single microband electrodes.

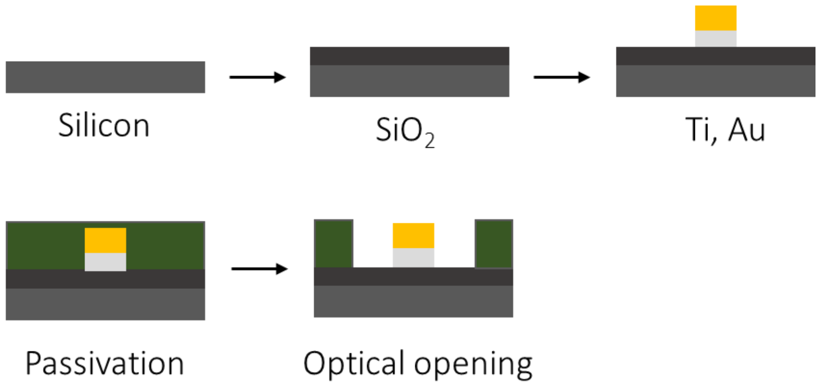

Figure S2. Schematic of the sensor fabrication process.

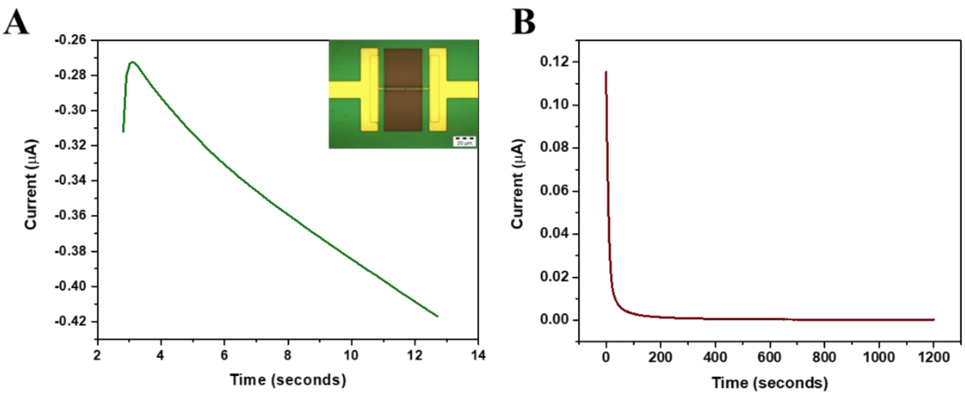

Figure S3. A) Typical amperometric deposition of Pt-B at -0.75V versus the on chip platinum reference electrode for 10 seconds with a quiet time of 2 seconds (inset shows microscopy image post electrodeposition) B) Typical amperometric deposition of 5 mM o-phenylenediamine with 2.5 mM β-cyclodextrin and 10mg/ml of Glucose Oxidase for 20 minutes.

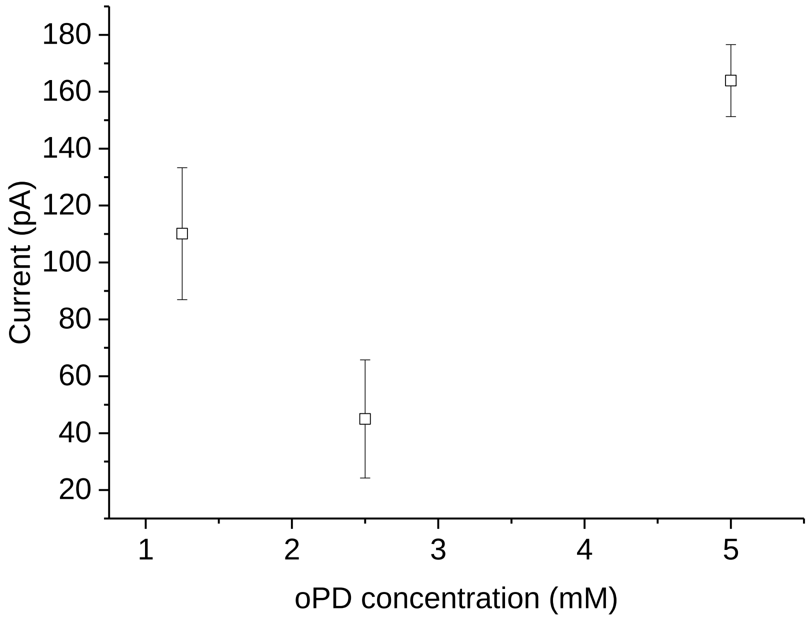

Figure S4. Current response for a 0.5mM glucose concentration at the Au/Pt-B/o-PD/β-cyclodextrin surface where the o-PD concentration was varied (1.25 mM, 2.5 mM and 5 mM) whilst maintaining GOx at 10 mg/ml and the β-cyclodextrin at 2.5 mM in 50 mM PB (pH 7.4).

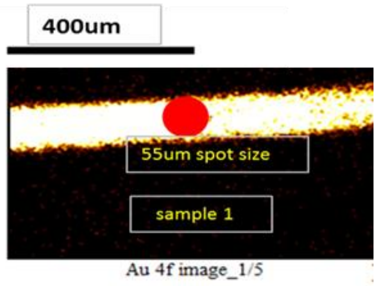

Figure S5. Minimum spot size for XPS analysis on a planar macroscopic band electrode.

Table S1. Quantification from XPS survey spectra at each modification.

Electrode layer	Atomic %						
	**O**	**C**	**N**	**Si**	**Au**	**Pt**	**Pb**
Bare Au	17.0	24.0	21.5	20.5	16.2	-	-
Pt-B	18.9	35.8	15.3	20.7	-	8.6	0.6
o-PD/β-cyclodextrin	17.3	51.9	17.9	12.7		0.2	-

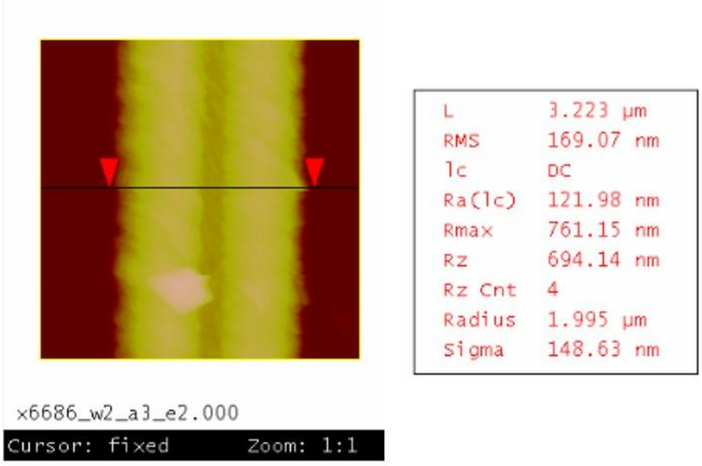

Figure S6. AFM section analysis of the Pt-B/o-PD/ β-cyclodextrin/GOx modified electrode surface.

Table S2. Table depicting change in current in the presence of interfering species.

**Interfering species**	**Concentration (mM)**	**(I/Io * 100)**
Ascorbic Acid	0.5	100.88 %
Uric Acid	0.5	99.38 %
Salycyclic Acid	0.5	104.44 %
Fructose	1	104.85 %
Mannose	1	104.58 %

*Where I is the current response of 2.5 mM Glucose in 50 mM PB(pH 7.4) and Io is the current response of 2.5 mM Glucose and the given concentration of the interferent in 50 mM PB (pH 7.4).

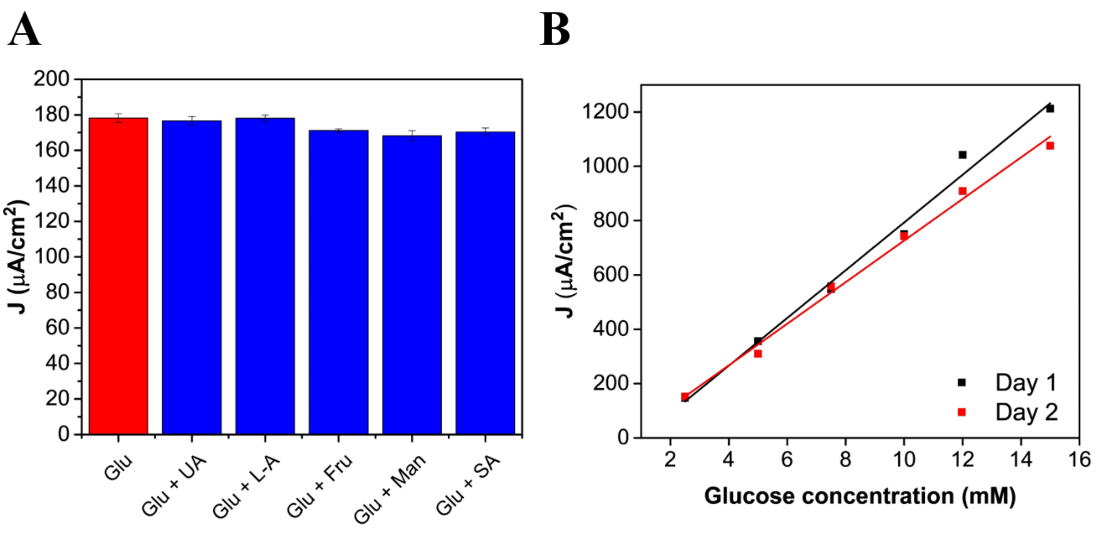

Figure S7. A) Bar chart depicting the background subtracted currents of a 2.5 mM glucose solution and that of a 2.5 mM glucose solution with other potential interfering species B) A calibration obtained at the same sensor at Day 1 and again at Day 2 after sensor preparation.
